# ^1^H NMR-Based Metabolomic Analysis of Sub-Lethal Perfluorooctane Sulfonate Exposure to the Earthworm, *Eisenia fetida*, in Soil

**DOI:** 10.3390/metabo3030718

**Published:** 2013-08-27

**Authors:** Brian P. Lankadurai, Vasile I. Furdui, Eric J. Reiner, André J. Simpson, Myrna J. Simpson

**Affiliations:** 1 Department of Chemistry, University of Toronto, 1265 Military Trail, Toronto, Ontario M1C 1A4, Canada; E-Mails: brian.lankadurai@mail.utoronto.ca (B.P.L); andre.simpson@utoronto.ca (A.J.S.); 2 Ontario Ministry of the Environment, 125 Resources Road, Toronto, Ontario M9P 3V6, Canada; E-Mails: vasile.furdui@ontario.ca (V.I.F.); eric.reiner@ontario.ca (E.J.R.)

**Keywords:** PFOS, metabolic profiling, mode of action, PFAAs, earthworms

## Abstract

^1^H NMR-based metabolomics was used to measure the response of *Eisenia fetida* earthworms after exposure to sub-lethal concentrations of perfluorooctane sulfonate (PFOS) in soil. Earthworms were exposed to a range of PFOS concentrations (five, 10, 25, 50, 100 or 150 mg/kg) for two, seven and fourteen days. Earthworm tissues were extracted and analyzed by ^1^H NMR. Multivariate statistical analysis of the metabolic response of *E. fetida* to PFOS exposure identified time-dependent responses that were comprised of two separate modes of action: a non-polar narcosis type mechanism after two days of exposure and increased fatty acid oxidation after seven and fourteen days of exposure. Univariate statistical analysis revealed that 2-hexyl-5-ethyl-3-furansulfonate (HEFS), betaine, leucine, arginine, glutamate, maltose and ATP are potential indicators of PFOS exposure, as the concentrations of these metabolites fluctuated significantly. Overall, NMR-based metabolomic analysis suggests elevated fatty acid oxidation, disruption in energy metabolism and biological membrane structure and a possible interruption of ATP synthesis. These conclusions obtained from analysis of the metabolic profile in response to sub-lethal PFOS exposure indicates that NMR-based metabolomics is an excellent discovery tool when the mode of action (MOA) of contaminants is not clearly defined.

## 1. Introduction

Perfluoroalkyl acids (PFAAs) are a class of anthropogenic chemicals that have been distributed globally, owing to their wide usage in many industrial and consumer-use applications [1,2,3]. PFAAs are lipophobic, hydrophobic, thermally stable, resistant to acids, bases and oxidizing agents and possess surfactant-like properties [2,3,4]. As such, the high chemical stability of PFAAs also results in long-term environmental persistence [1,3,4]. Perfluorooctane sulfonate (PFOS) is reported to be the most prevalent type of PFAA in the environment [2,5]. It is also the final breakdown product of many perfluorinated chemicals and has no known natural degradation pathway [3]. PFOS has been detected in human breast milk and blood serum, wildlife blood serum and livers and in fish [2,6,7]. Neonatal mortality, decreased body weight and size, increased liver weight, tumors in the pancreas, liver and testicles and changes to fatty acid metabolism were observed with PFOS exposure to rats, mice and monkeys [8,9,10]. On account of its environmental persistence and toxicity to organisms, PFOS has been recently added to Annex B of the Stockholm Convention on Persistent Organic Pollutants, which restricts the production of PFOS to a few specific applications [3,11].

PFOS has been detected in various soil environments [12,13,14,15,16]. Li *et al.* [16] reported PFOS concentrations around 10 μg/kg in soils collected from various sites in Shanghai, China. Sepulvado *et al.* [17] also detected PFOS in biosolid-amended agricultural soil from Chicago at concentrations close to 500 μg/kg. Das *et al.* [18] measured PFOS concentrations as high as 16 mg/kg in fire training areas that used aqueous film-foaming forms with PFOS as the active ingredient. However, toxicity studies involving soil dwelling invertebrates and plants are rare. Earthworms are often used as model organisms to monitor soil toxicity, because they are directly exposed to soil contaminants through ingestion and passive absorption [19,20]. Previous studies have examined the toxicity of PFOS to the earthworm, *Eisenia fetida*, in artificial soil and reported LC_50_ (the concentration that causes 50% mortality) values of 405 mg/kg and 365 mg/kg after seven and fourteen days of exposure, respectively [12,21]. Joung *et al.* [12] reported a no observable effect concentration (NOEC) in *E. fetida* based on mortality tests for PFOS soil concentrations of 160 mg/kg for both seven and fourteen days of exposure, whereas Sindermann *et al.* [21] observed an NOEC value of 77 mg/kg based on mortality tests in *E. fetida* after fourteen days of PFOS exposure. These studies illustrated that PFOS can be toxic to *E. fetida* and may pose a threat to soil quality and ecosystem health. In the environment, exposure to chemicals mostly occurs at sub-lethal or very low concentrations, which may also exert adverse physiological responses in many organisms [22,23]. Stubberud [24] conducted reproduction tests with *E. fetida* after PFOS exposure and reported EC_50_ (half maximal effective concentration) values of 103 mg/kg for number of cocoons, 80 mg/kg for number of juveniles and 29 mg/kg for the weight of juveniles. The NOEC value from the reproduction tests was reported as 10 mg/kg, which suggested that the reproduction tests were more sensitive indicators of PFOS exposure than the mortality tests and that adverse responses are being observed in the reproduction of *E. fetida* with sub-lethal exposure [24]. Although valuable, reproduction tests do not provide any detail regarding the toxic mode of action (MOA) of PFOS in earthworms. Analyzing the fluctuations in endogenous metabolite levels (such as amino acids and sugars) in response to sub-lethal contaminant exposure may provide insight regarding the MOA of the chemical [25,26]. Therefore, developing reproducible and high-throughput analytical methods that are capable of assessing organism responses to sub-lethal contaminant exposure may be indispensable.

^1^H nuclear magnetic resonance (NMR)-based metabolomics has emerged as a powerful tool for measuring organism responses to various types of environmental stressors [27,28]. Metabolomics involves measuring fluctuations in low molecular weight endogenous metabolite concentrations, such as sugars and amino acids, in response to a defined external stressor [27,28]. Nuclear magnetic resonance (NMR)-based earthworm metabolomics has shown promise as a rapid and reproducible technique that can elucidate the contaminant’s MOA and, also, identify potential metabolite indicators (or bioindicators) of exposure in response to sub-lethal contaminant exposure [29,30,31]. A previous work showed, for the first time, that ^1^H NMR-based metabolomics is able to distinguish between the responses of PFOS and perfluorooctanoic acid (PFOA) exposed *E. fetida* earthworms in contact tests [32]. We were also able to elucidate the MOA of both PFOS and PFOA in *E. fetida* after short-term exposure (48 hours). However, we only examined four exposure concentrations and, also, conducted filter paper contact exposure tests, which may not represent all of the complexities that are involved in soil exposure (*i.e.*, bioavailability). Therefore, further research needs to be conducted to better understand the responses of *E. fetida* to PFAA exposure in the environment.

In this study, ^1^H NMR-based metabolomics was used to investigate the response of the earthworm, *E. fetida*, after exposure to an artificial soil spiked with sub-lethal PFOS concentrations by exploring both concentration-dependent and time-dependent relationships. Brown *et al.* [33] and Whitfield Åslund *et al.* [34] showed that two-day exposure to sub-lethal concentrations of phenanthrene and polychlorinated biphenyl (PCB), respectively, elicited significant metabolic responses in *E. fetida*. Joung *et al.* [12] reported similar LC_50_ values for both the seven and fourteen-day PFOS exposures (405 mg/kg and 365 mg/kg, respectively). Therefore, to determine an appropriate exposure period and, also, to test the exposure time-response of *E. fetida* to PFOS, we chose exposure time lengths of two days, seven days and fourteen days of exposure recommended by the organization for economic co-operation an development (OECD) earthworm acute toxicity tests in artificial soil] [35]. Our first objective was to compare the metabolic response of *E. fetida* to PFOS exposure in artificial soil to the metabolic response of *E. fetida* in contact tests reported in Lankadurai *et al.* [32] and determine the appropriateness of contact tests in predicting soil exposure responses. We also tested if NMR-based metabolomics was capable of detecting significant perturbations in the metabolic profile at PFOS exposure concentrations lower than the NOEC-values obtained from traditional toxicity tests, such as mortality tests and reproduction tests [21,24]. Based on previous NOEC reports for PFOS, we chose six sub-lethal PFOS soil exposure concentrations ranging from 5 mg/kg to 150 mg/kg. The United States Environmental Protection Agency (US EPA) has set a residential soil screening level for PFOS of 6 mg/kg [36]. Therefore, we wanted to examine if there are significant metabolic responses at the lowest PFOS exposure concentration of 5 mg/kg. We also verified if changes in biochemical processes, such as increased fatty acid oxidation and perturbations in energy metabolism that were observed in other organisms and were proposed in Lankadurai *et al.* [32], are observable after soil exposure, as well [3,9]. This study will help assess if NMR-based metabolomics can be applied as a routine ecotoxicological tool for assessing the toxicity of PFOS in soil environments.

## 2. Results and Discussion

### 2.1. Multivariate Statistical Analysis

**Figure 1:**
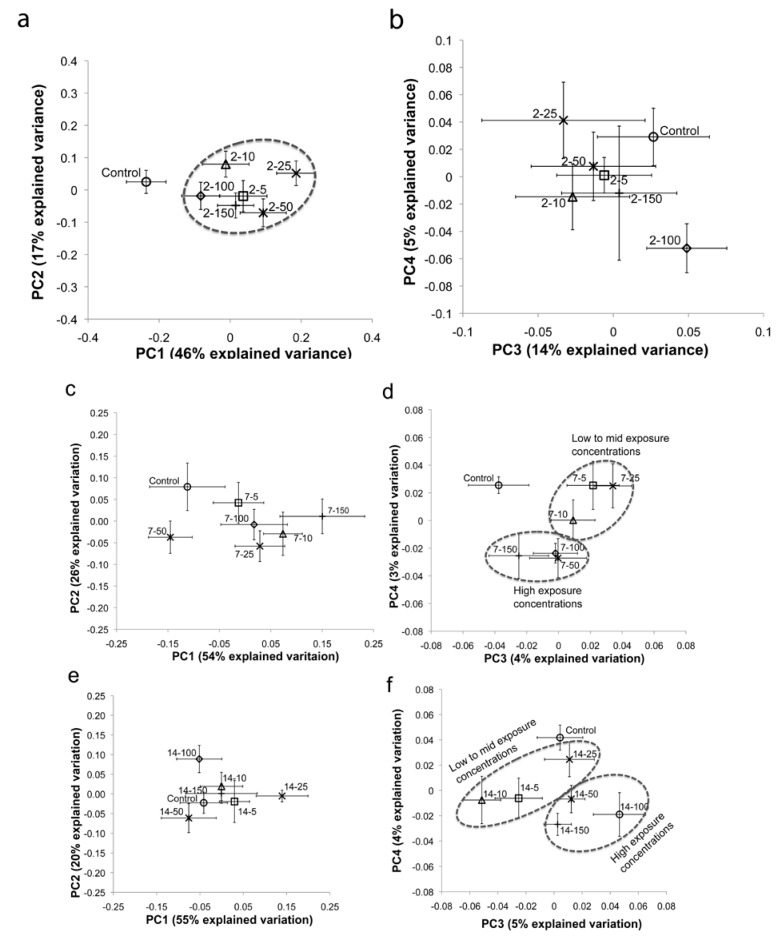
Average principal component analysis (PCA) score plot for the ^1^H NMR spectra of *Eisenia fetida* tissue extracts after perfluorooctane sulfonate (PFOS) exposure of two days, (**a**) PC1 (first PCA component) *versus* PC2 (second PCA component), (**b**) PC3 (third PCA component) *versus* PC4 (fourth PCA component), seven days, (**c**) PC1 *vs.* PC2, (**d**) PC3 *vs.* PC4, and fourteen days, (**e**) PC1 *vs.* PC2, (**f**) PC3 *vs.* PC4. The mean scores for the PFOS-exposed earthworms are denoted by the exposure length, followed by the corresponding exposure concentration (for example, 2–100 denotes a two-day exposure to 100 mg/kg of PFOS in Organization for Economic Corporation and Development soil). The mean scores (with associated standard error) were obtained by averaging the scores of the controls and each exposure concentration. The ellipses were constructed as visual aids.

**Figure 2:**
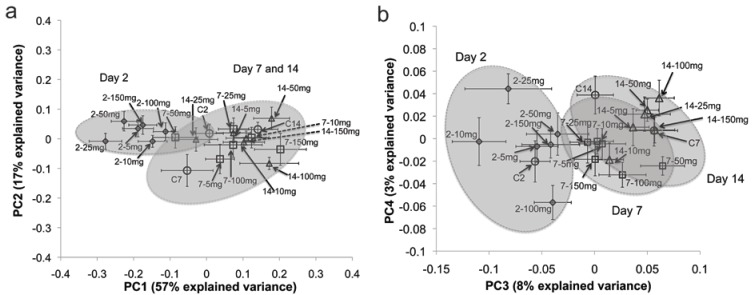
Average principal component analysis (PCA) score plot for the ^1^H NMR spectra of *Eisenia fetida* tissue extracts after PFOS exposure of two, seven and fourteen days. (**a**) PC1 (first PCA component) *versus* PC2 (second PCA component), (**b**) PC3 (third PCA component) *versus* PC4 (fourth PCA component). The mean scores for the PFOS-exposed earthworms are denoted by the exposure length, followed by the corresponding exposure concentration (for example, 2–50 denotes a two-day exposure to 50 mg/kg of PFOS in Organization for Economic Corporation and Development soil). The mean scores (with associated standard error) were obtained by averaging the scores of the controls and each exposure concentration for each day of exposure. The ellipses were constructed as visual aids.

**Figure 3:**
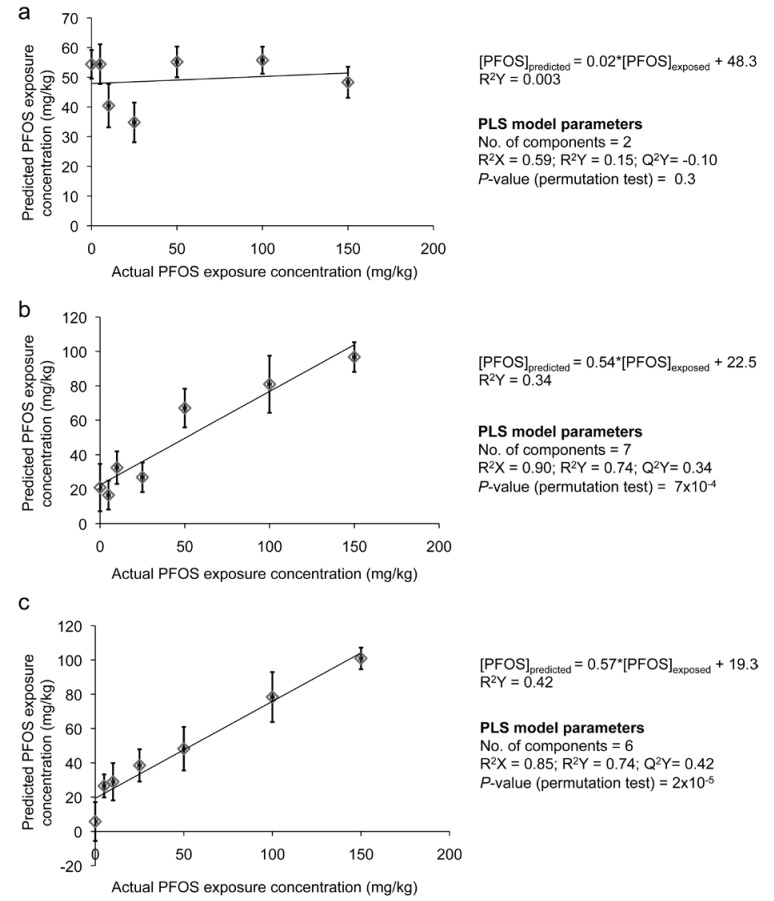
Average predictions of PFOS concentrations (ŷ_i_), given spectra i by the PLS model derived from the leave-one-out cross-validation procedure with spectra i omitted for PLS models constructed with the bucketed ^1^H NMR spectra as the X-table and the PFOS exposure concentrations as the Y variable. The solid line indicates a linear regression between the actual and predicted values. The PLS-regression models correspond to (**a**) two days of exposure, (**b**) seven days of exposure and (**c**) fourteen days of exposure. The error bars represent the standard error of the mean.

Average principal component analysis (PCA) score plots were constructed using the ^1^H NMR spectra of *E. fetida* tissue extracts to compare the metabolic response of the PFOS exposed earthworms to the control (unexposed) earthworms (Figure 1) [37,38,39]. The average PCA score plot (PC1 *vs.* PC2) for the two-day exposure period showed clear separation between controls and PFOS-exposed earthworms (Figure 1a). However, the separation of the PFOS-exposed earthworms from the controls did not reveal a clear concentration-dependent trend. Lankadurai *et al.* [32] illustrated a concentration-dependent separation from the controls in the PCA score plot (PC1 *vs.* PC2) after PFOS exposure to *E. fetida* via contact tests. Therefore, we investigated PC3 and PC4 scores, as well, to determine if they reveal concentration-dependent trends. The PC3 *vs.* PC4 score plot also illustrated separation of PFOS-exposed earthworms from the controls, but the extent of the separation was not dependent on the exposure concentration. However, the separation in the PC3 *vs.* PC4 score plot was not as pronounced as was observed with PC1 *vs.* PC2 (Figure 1b). The average PCA score plot (PC1 *vs.* PC2) for the seven-day exposure period also revealed clear separations between controls and exposed earthworms that were not dependent on the exposure concentrations (Figure 1c). However, the PC3 *vs.* PC4 scores plot for the seven-day exposure illustrated that the higher exposure concentrations (50, 100 and 150 mg/kg) were more separated from the controls compared to the lower exposure concentrations (five, 10 and 25 mg/kg; Figure 1d). The average PCA score plot (PC1 *vs.* PC2) for the fourteen-day exposure period did not illustrate a clear separation from the controls for all of the exposure concentrations (Figure 1e). Nevertheless, the PC3 *vs.* PC4 average score plot revealed that the higher exposure concentrations (50, 100 and 150 mg/kg) were better separated from the controls compared to the low and mid-exposure concentrations (five, 10 and 25 mg/kg; Figure 1f). Overall, the PC1 *vs.* PC2 (explained 63% of the variation in the metabolic response) score plot showed better separation (not dependent on exposure concentration) from the controls for the two-day exposure period, whilst the PC3 *vs.* PC4 (explained about 9% of the variation in the metabolic response) score plots showed better (concentration-dependent) separations from the controls for the seven and fourteen-day exposures. This presents two interesting conclusions regarding the exposure time-dependent response of *E. fetida* to PFOS exposure: Firstly, exposure to PFOS for two days elicited a strong response by the earthworms that is independent of the exposure concentration and one that dominates the variation observed in the metabolic profile (based on PC1 *vs.* PC2 score plot). Secondly, longer exposures seem to demonstrate a different MOA that is exposure concentration-dependent and one that does not dominate the observed variation in the metabolic profile (based on PC3 *vs.* PC4 score plots). Longer exposure periods lead to prolonged starvation in both controls and exposed earthworms. Therefore, the overall variation in the metabolic profile of *E. fetida* may be dominated by starvation responses with longer exposure periods, which may explain the reduced separations from the controls at the seven- and fourteen-day exposure periods in the PC1 and PC2 score plots (Figure 1c,e). The average score plot (both PC1 *vs.* PC2 and PC3 *vs.* PC4) summarizing all the exposure days and exposure concentrations (Figure 2) reveals that the scores of the seven- and fourteen-day exposures are clustered together, whereas the scores of the two-day exposure are separated from the seven- and fourteen-day exposures. This also indicates that the seven- and fourteen- day PFOS exposures activate a MOA that is different from the response of *E. fetida* after two days of exposure. Partial least squares regression (PLS)-regression models were constructed to ascertain the strength and significance of the relationship between the metabolic profile and the PFOS exposure concentration (Figure 3 and Supplementary Material, Table S1 and Figure S3) [34,40,41]. The PLS-regression model for the two-day exposure had no apparent predictive power, as illustrated by a negative Q^2^Y value (cross-validated PLS-regression with two components, R^2^X = 0.59, R^2^Y = 0.15, Q^2^Y = ˗0.10, *p* = 0.3; Figure 3 and Supplementary Material, Table S1). However, the PLS-regression model for the seven-day exposure suggested a weak, but significant, linear correlation between the *E. fetida* metabolic profile and the PFOS exposure concentration (cross-validated PLS-regression with seven components, R^2^X = 0.90, R^2^Y = 0.74, Q^2^Y = 0.34, *p* = 7 × 10^−4^). The fourteen-day exposure produced a PLS-regression model that had the best predictive power and strongest linear relationship between the metabolic profile and the PFOS exposure concentration (cross-validated PLS-regression with six components (R^2^X = 0.85, R^2^Y = 0.74, Q^2^Y = 0.42, *p* = 2 × 10^−5^). Similar to the PCA analysis, the PLS-regression models also suggested that two separate MOAs may be operational, one at the shorter exposure time of two days that is exposure concentration-independent and the other at the longer exposure times of seven and fourteen days that is concentration-dependent (Figure 3). The clear separation observed between the controls and PFOS-exposed earthworms even at the very low exposure concentration of 5 mg/kg in the PCA score plots and the significant linear correlation between the metabolic profile and the PFOS exposure concentration observed in the PLS-regression analysis for the seven and fourteen-day exposures suggests that NMR-based metabolomics is a much more sensitive indicator of PFOS exposure than the traditional toxicity tests, such as mortality (NOEC = 77 mg/kg) [21] and reproduction (NOEC = 10 mg/kg) [42] tests. In addition, we also observed clear separation from controls at the lower exposure of 5 mg/kg, which was below the residential soil screening level for PFOS (6 mg/kg) set by the US EPA [36].

### 2.2. Metabolic Changes in Response to PFOS Exposure

PCA loading plots were constructed to determine the metabolites that were responsible for the separation between the controls and PFOS-exposed earthworms in the PCA scores plots (Supplementary Material, Figures S1 and S2). In addition, *t*-test-filtered ^1^H NMR difference spectra were also constructed in an exploratory capacity to identify metabolites that increased or decreased significantly (at α = 0.05) relative to the controls (Supplementary Material, Figures S4–S6) [31,43,44]. The PCA loading plots and the *t*-test-filtered ^1^H NMR difference spectra identified the sugars, maltose (5.41 ppm) and glucose/maltose (5.23 ppm), Krebs cycle intermediates, succinate (2.39 ppm), fumarate (6.51 ppm) and malate (2.37 ppm), amino acids, leucine (0.95 ppm), valine (1.03 ppm), alanine (1.47 ppm), arginine (1.91 ppm), glutamate (2.35 ppm), lysine (3.01 ppm), glycine (3.55 ppm) and phenylalanine (7.31 ppm), messenger molecules, *scyllo*-inositol (3.35 ppm) and *myo*-inositol (4.05 ppm), the osmolyte, betaine (3.25 ppm), the energy molecule, adenosine triphosphate (ATP; 8.23 ppm), short-chain fatty acids (1.27 ppm for -CH_2_ and 0.83 ppm for -CH_3_) and 2-hexyl-5-ethyl-3-furansulfonate (HEFS; 1.27 ppm for -CH_2_, 0.83 and 1.17 ppm for -CH_3_, 6.17 ppm for -CH from the furan ring) as the metabolites that significantly fluctuated in their concentrations in response to PFOS exposure (Supplementary Material, Figures S1, S2, S4–S6). Both the loading plots and the difference spectra identified that the 3.40–4.00 ppm region of ^1^H NMR spectra, which contains overlapping resonances from sugars and amino acids, increased or decreased in response to PFOS exposure. However, these signals cannot be clearly assigned to individual metabolites, due to overlapping resonances within this region. The loading plots for PC1 and PC2 (Supplementary Material, Figure S1a, b and e) for the two, seven and fourteen days of exposure illustrated that betaine and HEFS were the major contributors to the variation in the metabolic response and had the greatest influence on the separation observed between the controls and the exposed earthworms in the PCA score plots (Figure 1a,c,e). The PC1 *vs.* PC2 score plots did not reveal any concentration-dependent separations for any of the exposure lengths, suggesting that the fluctuations in betaine and HEFS are also exposure concentration-independent. The PC3 and PC4 loadings plots for the two, seven- and fourteen-day exposures showed that *scyllo*-inositol, alanine, glutamate and leucine also had substantial contributions to the metabolic variation in addition to betaine and HEFS (Supplementary Material, Figure S1b, d, f). It was also interesting to note that the decreased contributions of HEFS and betaine in the PC3 and PC4 loading plots for the seven and fourteen day exposures were correlated with better concentration-dependent separations from the controls in the PC3 *vs.* PC4 score plots (Figure 1d, f) compared to the PC1 *vs.* PC2 score plots (Figure 1c ,e). This illustrated that the responses of the metabolites, other than betaine and HEFS, to PFOS exposure is largely responsible for the observed concentration-dependent patterns in the PC3 *vs.* PC4 score plots for the seven- and fourteen-day exposures.

**Figure 4:**
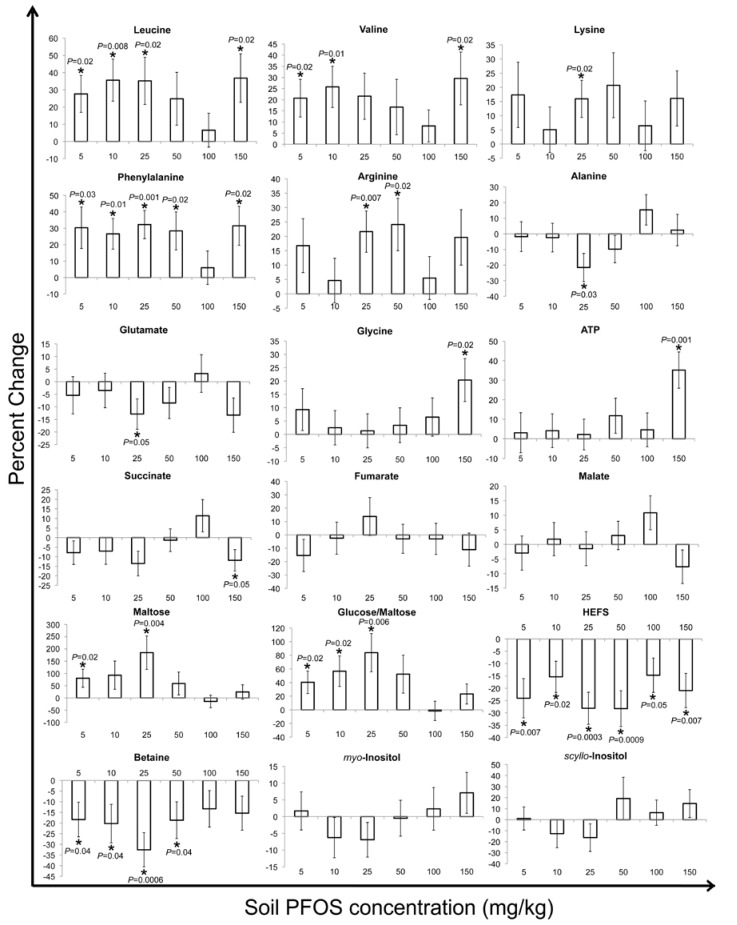
Percent (%) change in selected metabolites of two-day PFOS exposed tissue extracts compared with the control earthworms. The % changes in the intensity of *Eisenia fetida* metabolite resonances of exposed earthworms relative to the controls were obtained by first subtracting the buckets that pertain to the metabolites in the control earthworms from the exposed earthworms for each exposure concentration and then dividing again by the buckets in the control earthworms [43]. The percent changes that were significantly (at α = 0.05) different from the control (based on a *t*-test (two tailed, equal variances) of control *vs.* exposed) are labeled with an asterisk (*) and also show the corresponding *p*-values. The percent changes are shown with their associated standard error.

**Figure 5:**
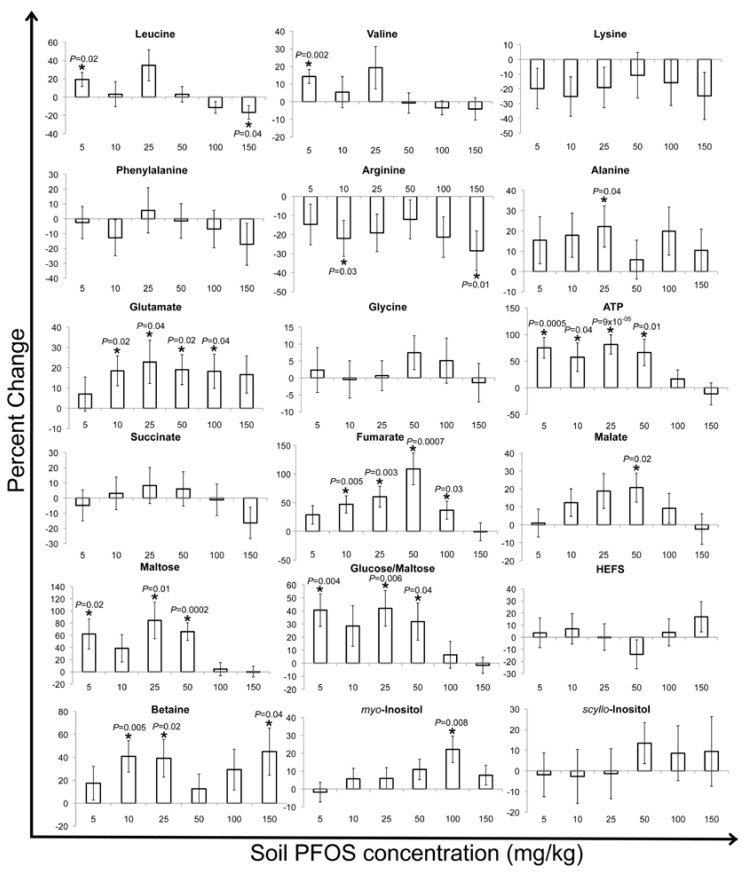
Percent (%) change in selected metabolites of seven-day PFOS-exposed *Eisenia fetida* tissue extracts compared with the control earthworms. The % changes in the intensity of metabolite resonances of exposed earthworms relative to the controls were obtained by first subtracting the buckets that pertain to the metabolites in the control earthworms from the exposed earthworms for each exposure concentration and then dividing again by the buckets in the control earthworms [31,43]. The percent changes that were significantly (at α = 0.05) different from the control (based on a *t*-test (two tailed, equal variances) of control *vs.* exposed) are labeled with an asterisk (*) and also show the corresponding *p*-values. The percent changes are shown with their associated standard error.

**Figure 6:**
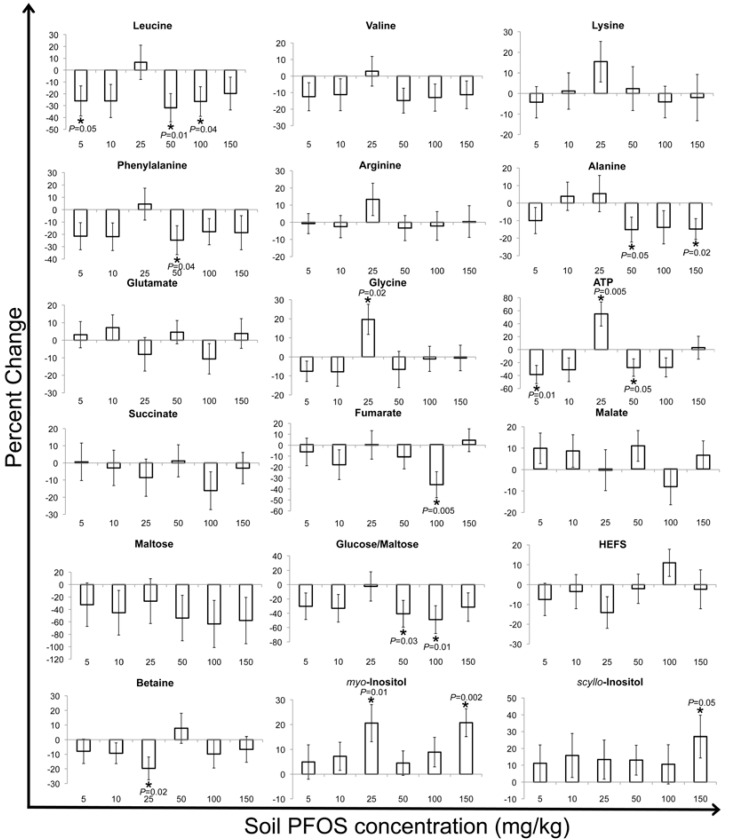
Percent (%) change in selected metabolites of fourteen-day PFOS-exposed *Eisenia fetida* tissue extracts compared with the control earthworms. The % changes in the intensity of metabolite resonances of exposed earthworms relative to the controls were obtained by first subtracting the buckets that pertain to the metabolites in the control earthworms from the exposed earthworms for each exposure concentration and then dividing again by the buckets in the control earthworms [31,43]. The percent changes that were significantly (at α = 0.05) different from the control (based on a *t*-test (two tailed, equal variances) of control *vs.* exposed) are labeled with an asterisk (*) and also show the corresponding *p*-values. The percent changes are shown with their associated standard error.

The percent changes in discernible metabolite bucket intensities were examined to determine the fluctuations after PFOS exposure and, also, to delineate the MOA (Figure 4, Figure 5, Figure 6) [26,30,43,45]. In general, the percent changes in the metabolite concentrations relative to the controls did not reveal any consistent concentration-dependent patterns after two, seven or fourteen days of exposure. This was in contrast to what was observed in a previous study in which *E. fetida* were exposed to PFOS via the filter paper contact test after two days of exposure [32]. This difference in the observed response of *E. fetida* between the contact and soil exposure studies reveals the complexity in the exposure routes of contaminants in soil as compared to a simple filter paper contact test. In the present study, leucine, valine, lysine, phenylalanine and arginine revealed significant (at α = 0.05) increases after two days of soil exposure to PFOS (Figure 4). After seven and fourteen days of exposure, the above-mentioned amino acids showed varying responses (Figure 5, Figure 6). In Lankadurai *et al.* [32], leucine, valine, lysine, phenylalanine and arginine decreased significantly (at α = 0.05) at all exposure concentrations in the contact filter paper test. This was attributed to the production of enzymes involved in fatty acid oxidation, which would have resulted in a decrease in these free amino acids. Binding of PFOS to the peroxisome proliferator-activated receptor alpha (PPARα), a mammalian nuclear hormone receptor involved in lipid and lipoprotein metabolism, results in increased peroxisome production and a heightened oxidation of fatty acids [46,47]. The nuclear hormone receptor involved in lipid metabolism has not been identified in *E. fetida*. However, the nuclear hormone receptor-49 *(nhr-49)* is involved in regulating lipid metabolism in the nematode worm, *Caenorhabditis elegans*, much like PPARα in mammals [48,49].

Therefore, this led to the hypothesis that *E. fetida* also possesses nuclear hormone receptors similar to the *nhr-49* that may be activated by the binding of PFOS, leading to an initiation of fatty acid oxidation and the subsequent decrease in amino acids, due to the production of enzymes involved in β-oxidation [32]. Analyzing the percent changes in these amino acids over the various exposure lengths tested in this study revealed that as the exposure time increased, the amino acid concentrations generally tend to decrease in the exposed earthworms relative to the controls (Figure 4, Figure 5, Figure 6). The percent increase in these amino acids that was observed after two days of exposure is similar to what was observed in *E. fetida* after phenanthrene exposure [25,50]. Exposure to phenanthrene elicits a non-polar narcosis-type mechanism [50,51,52]. PFOS, due to its surfactant-like properties, disrupts biological membrane structure [53,54,55]. The osmolyte, betaine, and HEFS, a compound that is specific to earthworms and has been postulated to be involved in membrane stabilization [30,39], also significantly decreased after two days of exposure (Figure 4). This was in contrast to PFOS exposures by contact tests, where both HEFS and betaine significantly increased after exposure, showing that different modes of exposure have varying MOAs in the disruption of membranes [32]. Betaine and HEFS also dominated the PC1 and PC2 loading plot for the two-day exposure (Supplementary Material, Figure S1a) and, therefore, contributed the most to the clear separation observed between the controls and exposed earthworms in the PC1 *vs.* PC2 score plot (Figure 1a). The significant fluctuations in betaine and HEFS after PFOS exposure may be as a response to counteract the disruption in the membrane structure brought about by PFOS. Therefore, we hypothesize that after two-days of PFOS exposure in soil, there is a non-polar narcosis type MOA in *E. fetida*. The general decrease in these amino acids with exposure times > 2 days was similar to what was observed with PFOS exposure via contact filter paper tests [32]. This suggests that fatty acid oxidation may have been initiated by PFOS with longer exposures.

PFOS exposure resulted in a significant (at α = 0.05) concentration-dependent decrease of ATP (the energy currency of the cell) relative to the controls in contact tests [32]. This was ascribed to the interruption of ATP synthesis by PFOS disrupting the structure of the inner mitochondrial membrane, thereby increasing its permeability and altering the proton (H^+^) gradient required for the functioning of the ATP synthase enzyme [55,56,57]. However, ATP concentrations increased relative to the controls after phenanthrene exposure to *E. fetida* via contact tests, as was observed after the two-day and seven-day exposures in our current study [25,41,45]. As the exposure time increased to fourteen days, ATP significantly decreased relative to the controls in the PFOS-exposed earthworms (Figure 6). Therefore, the disruption of the inner mitochondrial membrane structure by PFOS and the consequent altering of the proton (H^+^) gradient and interruption of ATP synthesis may only take place after PFOS exposure that is longer than seven days in soil. The sugars, maltose and glucose/maltose, generally increased significantly (at α = 0.05) relative to the controls at PFOS exposure concentrations ≤50 mg/kg for the two- and seven-day exposures (Figure 4, Figure 5). After fourteen days of exposure, maltose and glucose/maltose generally decreased relative to the controls, with significant (at α = 0.05) decreases at the 50 and 100 mg/kg PFOS exposure concentrations for glucose/maltose (Figure 6). Significant decreases were observed in maltose and glucose/maltose after PFOS exposure in contact tests [32]. Maltose also decreased significantly (at α = 0.05) after phenanthrene exposure in both contact and soil exposure tests [25,33,41,45].

The decrease in maltose and glucose was attributed to the increase in glycolysis, due to an enhanced energy requirement brought about by the organisms attempt to counteract the toxicity of the xenobiotic. In this study, the significant increases in maltose and glucose/maltose were also correlated with a significant increase in ATP (Figure 4, Figure 5, Figure 6). This may be due to a feedback loop, which restricts glycolysis, due to the accumulation of ATP [58]. As exposure time increased to fourteen days and ATP concentrations began to decrease significantly, probably due to a disruption of ATP synthase function, glycolysis is enhanced, and maltose and glucose begin to decrease significantly. 

The contact test study by Lankadurai *et al.* [32] identified significant (at α = 0.05) increases in both succinate and malate in response to PFOS exposure, but did not observe any significant changes in fumarate concentrations. Although the percent changes in these Krebs cycle intermediates did not reveal any clear patterns in the present study (Figure 4, Figure 5, Figure 6), the significant (at α = 0.05) changes that were observed may reflect the altered expression of genes involved in producing enzymes for the Krebs cycle that were reported in previous studies of PFOS exposure to rats and humans [59,60,61]. Glutamate only decreased significantly at the 25 mg/kg exposure concentration after two days of exposure (Figure 4). After seven days of exposure, glutamate generally increased significantly (at α = 0.05) relative to the controls (Figure 5), whilst fourteen days of exposure did not illustrate any significant changes in glutamate concentrations relative to controls (Figure 6). Glutamate was also shown to increase significantly after PFOS exposure for two days via contact tests [32]. A disruption of the regular functioning of the Krebs cycle due to PFOS exposure was accredited as a possible reason for an increased conversion of α-ketoglutarate (a Krebs cycle intermediate) to glutamate via the glutamate dehydrogenase enzyme [58]. Interestingly, the significant accumulation of fumarate after seven days of exposure was correlated with a significant increase in glutamate, suggesting a possible feedback mechanism, resulting in an increased conversion of α-ketoglutarate to glutamate (Figure 6). 

The concentrations of the inositol isomers (*myo* and *scyllo*-) did not reveal consistent trends in their fluctuations to PFOS exposure, but did show significant (at α = 0.05) increases for some exposure concentrations after the seven and fourteen days of exposure (Figure 4, Figure 5, Figure 6). Lankadurai *et al.* [32] also illustrated a significant increase in *myo*-inositol at high PFOS exposure concentrations in contact tests. Significant (at α = 0.05) increases in glycine were only observed at the 150 mg/kg exposure concentration after two days of exposure and at the 25 mg/kg exposure concentration after fourteen days of exposure (Figure 4, Figure 5, Figure 6). The inositol isomers and glycine function as osmolytes, and the significant increases observed may be a means to cope with the changes in osmotic pressure brought about by a disruption in the membrane structure, due to the surfactant-like properties of PFOS [62,63,64].

## 3. Experimental Section

### 3.1. Soil Spiking and Total Soil PFOS Concentrations

An artificial soil medium was prepared by mixing sphagnum peat (Ward’s Natural Science), Kaolin clay (Ward’s Natural Science) and sand (Ward’s Natural Science) in a 1:2:7 ratio, as described by the OECD Earthworm Acute Toxicity test protocol [35]. Initially, 125 g (dry weight) of the artificial soil was added to twenty-one 1 L clear glass jars. Ten millilitres of PFOS of 250, 500, 1,250, 2,500, 500 and 7,500 mg/L (heptadecafluorooctane sulfonic acid potassium salt; 98%, Sigma-Aldrich) dissolved in acetone (HPLC grade, Fisher Scientific) were used to spike the soils of the six PFOS-exposed treatments for the two-, seven- and fourteen-day exposure classes. The unexposed control treatment soil was treated with 10 mL of acetone only. The soils were then left in the fume hood for 16 h to allow the acetone to evaporate [65]. An additional 375 g (dry weight) of soil was mixed thoroughly into each jar for a total of 500 g (dry weight) of soil per jar, resulting in total soil PFOS concentrations of 5, 10, 25, 50, 100 and 150 mg/kg (dry weight) for the PFOS-exposed treatments. Deionized water was used to wet the soils to a moisture content of 35% of soil dry weight, and the soils were allowed to absorb the water for 24 h before introducing earthworms into the jars [35]. The PFOS concentrations in the spiked soils were confirmed by extraction and quantification via liquid chromatography/mass spectrometry (see Supplementary Material, Section S2 for the methods) following the two-day, seven-day and fourteen-day exposure of the earthworms to the soils and found no evidence of degradation of PFOS during the experiment (data not shown).

### 3.2. Earthworm Exposure and Tissue Extraction

Ten matured earthworms with a visible clitellum were added to each of the six PFOS-spiked soils and the control soil. Initial average mass of the earthworms was 400 ± 5 mg (standard error) wet weight. There was no significant difference in the initial earthworm mass between the different treatment groups (ANOVA, F_6,245_ = 0.501, *p* = 0.8). Earthworms were kept in closed jars for the duration of the exposure period in natural light, after which the earthworms were removed from the soils and were rinsed with distilled water to remove soil particles. They were then depurated individually for 96 h on damp filter paper. The filter paper was changed every 24 h. Earthworms were then flash-frozen in liquid nitrogen, lyophilized, reweighed and stored frozen until extraction. 

The lyophilized earthworms were homogenized individually while in ice in a 1.5 mL centrifuge tube using a 5 mm-wide stainless steel spatula [66]. The homogenized earthworm tissue was then extracted using the two step methanol, water, chloroform tissue extraction protocol [67]. Ice-cold methanol (4 mL/g of earthworm dry weight) and ice cold water (0.85 mL/g of earthworm dry weight) were added to the tissue and vortexed for 15 seconds using a VX 100 vortexer (Labnet, NJ, USA). Chloroform (4 mL/g of earthworm dry weight) and water (2 mL/g of earthworm dry weight) were then added and vortexed for 60 seconds. The samples were then kept on ice for 10 minutes to allow partitioning between the polar and non-polar layers. The tissue mixture was centrifuged for 10 minutes at 12,000 rpm (~11, 000 g) using an International Equipment Company 21000 centrifuge (Fisher Scientific, Whitby, ON, Canada). The upper polar layer and the bottom non-polar layer were removed carefully into a 1.5 mL centrifuge tube and a 1.8 mL glass vial, respectively. Previous studies have reported that the polar fraction is more informative than the non-polar fraction regarding the metabolic perturbations of *E. fetida* in response to phenanthrene exposure using ^1^H NMR metabolomics [25,41]. Preliminary analysis of the non-polar fraction using high-resolution mass spectrometry (MS) did not detect any significant fluctuations in the lipid metabolic profile. Therefore, for a rapid and consistent analysis of the metabolic response of *E. fetida* to PFAA exposure, only the polar fraction was analyzed in this study. The polar fraction was dried under a constant nitrogen flow and was then reconstituted in 750 μL of a 0.2 M monobasic sodium phosphate buffer solution (NaH_2_PO_4_·2H_2_O; 99.3%; Fisher Chemicals, Whitby, ON, Canada) containing 0.1% (w/v) sodium azide (99.5% purity; Sigma Aldrich, Oakville, ON, Canada) as a preservative [68]. Buffer solution was made with D_2_O (99.9% purity, Cambridge Isotope Laboratories, Andover, MA, USA) and adjusted to a pD of 7.4 using NaOD (30% w/w in 99.5% D2O; Cambridge Isotope Laboratories Inc., Andover, MA, USA). The buffer solution also contained 10 mg/L of 2,2-dimethyl-2-silapentane-5-sulfonate sodium salt (DSS; 97%, Sigma Aldrich, Oakville, ON, Canada) as an internal standard [33,37]. The extract was vortexed for 30 seconds and, then, centrifuged at 12,000 rpm (~11,000 × g) for 10 minutes, and the supernatant was transferred into a new 1.5 mL centrifuge tube. Samples were then transferred into 5 mm High Throughput^plus^ NMR tubes (Norell Inc., NJ, USA) for ^1^H-NMR analysis.

### 3.3. ^1^H-NMR Spectroscopy

^1^H NMR spectra of the earthworm extracts were acquired with a Bruker Avance III 500 MHz spectrometer using a ^1^H-^19^F-^15^N-^13^C 5 mm Quadruple Resonance Inverse (QXI) probe fitted with an actively shielded Z gradient. ^1^H NMR experiments were performed on the polar fraction using Presaturation Using Relaxation Gradients and Echoes (PURGE) water suppression and 128 scans, a recycle delay of 3 s and 16 K time domain points [69]. Spectra were apodized through multiplication with an exponential decay corresponding to 0.3 Hz line broadening in the transformed spectrum and a zero filling factor of 2. All spectra were manually phased and calibrated consistently. The ^1^H NMR spectra were calibrated to the nine identical methyl protons of the trimethylsilyl group of the DSS internal reference (0.00 ppm).

### 3.4. Data and Statistical Analysis

The chemical range between 0.5 and 10 ppm represented all ^1^H NMR resonances in extracts and were divided into buckets that were 0.02 ppm in width using the AMIX 3.9.7 statistics tool for a total of 475 buckets (Bruker BioSpin, Rheinstetten, Germany) [25,45,70]. The area between 4.70–4.85 ppm was excluded to eliminate the small residual H_2_O/HOD signals for the polar fraction [25,32,45,71]. The integration mode was set at the sum of intensities, and the spectra were scaled to total intensity. This created a matrix in which each row represents an earthworm sample and each column contains the integrated area of the original spectral intensities contained within each bucket region. PCA score plots were generated individually for the different days of exposure, and a PCA score plot was also constructed, which compared all the treatment classes together. The scores from the PCA score plots were then imported into Microsoft Excel (version 12.0.6504, Microsoft Corporation, Redmond, WA, USA), were averaged per class (exposure concentration) and re-plotted with their associated standard errors. Corresponding PCA loadings plots, which show the relative weight for each bucket, were also acquired for each of the PCA score plots to identify the metabolites that were contributing to the separation between the scores of the control and exposed earthworms [33,37,39]. 

PLS-regression models were constructed to determine the relationship between the metabolic profile and PFOS exposure concentration after each day of exposure [34,40]. PLS-regression analyses were performed in [72] using the Chemometrics package [73] on the buckets generated by the AMIX 3.9.7 statistics tool, which represent the binned ^1^H NMR spectra. PLS-regression was performed via the non-linear iterative partial least squares (NIPALS) algorithm using PFOS concentration as the Y (response) matrix and the normalized bucket intensities from the ^1^H NMR spectra of all the earthworms as the X matrix of multiple predictors [34,41]. PLS models were cross validated using the leave-one-out cross validation [34,74,75]. The single cross validation (1CV) strategy [75] was used to determine the optimal number of components for each final PLS model. The explained variation of X (R^2^X) and Y (R^2^Y) were obtained for each PLS model as a measure of how well the model fit the training data [40]. The cross-validated R^2^Y value (denoted as Q^2^Y) was used as a preliminary measure of the predictive ability of the PLS model [34,74]. Response permutation testing was conducted to assess the significance of each PLS model [34,76,77]. This method consisted of keeping the X matrix (normalized binned ^1^H NMR spectra) constant, while randomly permuting the order of the PFOS exposure concentrations (Y matrix) 400 times. For each permutation, a new PLS model was fitted, and the Q^2^Y was calculated, providing a reference distribution of the Q^2^Y statistic. The significance of the original PLS model and the confidence in its validity is increased if its Q^2^Y value is higher than the values obtained for all of the PLS models built during the permutation tests [40].

Difference class ^1^H NMR spectra were constructed to identify metabolites that had significantly increased or decreased relative to the control [31,43,45]. The buckets generated by the AMIX 3.9.7 statistics tool, which represents the binned ^1^H NMR spectra of *E. fetida* extracts, were then imported into Microsoft Excel. A *t*-test (two-tailed, equal variances) was then performed comparing the buckets of the controls with that of the exposure class to identify the buckets that were statistically different at α = 0.05. Average ^1^H NMR spectra were obtained by averaging the buckets of each exposure concentration and control treatments separately. Difference class ^1^H NMR spectra were then obtained by subtracting the buckets of the average controls from that of the average exposure treatments. The buckets representing the resonances of metabolites that were not statistically significant from the controls were then replaced with a zero, resulting in a *t*-test filtered ^1^H NMR difference spectrum [41,43]. The buckets were then imported into Origin 7 (version 7.0383, OriginLab Corporation, Northampton, MA) to plot the difference ^1^H NMR spectra. The percent changes in the intensity of metabolite resonances of exposed earthworms relative to the control were obtained by dividing the buckets that pertain to the metabolites in the exposed ones by the corresponding buckets in the control. The metabolite resonances in the ^1^H NMR spectra of the earthworm tissue extracts were identified by comparing to previously published assignments [31,39,43,66,68,78]. 

## 4. Conclusions

Our study suggests that ^1^H NMR-based metabolomics is able to distinguish between the responses of PFOS-exposed and control (unexposed) earthworms at sub-lethal or very low exposure concentrations. Multivariate statistical analysis identified that the longer exposures of seven and fourteen days revealed a concentration-dependent metabolic response. A comparison of *E. fetida* responses to PFOS exposure in soil in this study and to PFOS exposure in contact tests [32] shows that contact tests elicited much more significant and consistent responses after two days of exposure. The MOA identified from soil exposure also appears to be more complex, because we initially observed a non-polar narcosis type mechanism after two days of exposure and, then, observed an increase in fatty acid oxidation after seven and fourteen days. In contrast, increased fatty acid oxidation was observed in contact tests after only two days [32]. This comparison illustrates that the modes of exposure of PFOS in soil and contact tests are clearly different. Sorption of PFOS to soil [79] may result in a decrease in its availability to the earthworms. Furthermore, the movement of earthworms within the soil, compared to *E. fetida* being placed on a filter paper applied with PFOS in contact tests, may decrease the amount of exposure through limited bioavailability in soil. Therefore, these results suggest that although contact tests can be used as a rapid method for determining the responses of earthworms to contaminants, soil exposure tests are required for an accurate assessment of the MOA. However, previous studies that exposed *E. fetida* to phenanthrene via contact and soil exposures observed similar MOAs by both methods after two days of exposure [33,37]. Hence, response of *E. fetida* to contact and soil exposure routes seems to be contaminant-specific. Increased fatty acid oxidation and disruption of biological membranes that were observed due to PFOS exposure conform to the hypothesized MOA [8,55,57,80]. ^1^H NMR-based metabolomics appears to be a more sensitive indicator of PFOS exposure than the traditional mortality tests and reproduction tests, which identified 77 mg/kg and 10 mg/kg, respectively, as the non-observable effect concentrations [21,24]. We also observed significant responses at the lower exposure of 5 mg/kg, which was below the residential soil screening level for PFOS (6 mg/kg) set by the US EPA [36]. Our study highlights the potential for NMR-based metabolomics to be used as a routine tool in ecotoxicological assessment of contaminated sites.

## Supplementary Files

Supplementary File 1 Supplementary (PDF, 478 KB)
