# Supplementary material for: 1H NMR-Based Metabolomic Analysis of Sub-Lethal Perfluorooctane Sulfonate Exposure to the Earthworm, Eisenia fetida, in Soil
Source: Metabolites. 2013 Aug 27;3(3):718–40. doi: 10.3390/metabo3030718 (PMC3901287; doi:10.3390/metabo3030718)

Supplementary Material

^{1}H NMR-Based Metabolomic Analysis of Sub-Lethal Perfluorooctane Sulfonate Exposure to the Earthworm *Eisenia fetida* in Soil

Brian P. Lankadurai [1], Vasile I. Furdui [2], Eric J. Reiner [2], André J. Simpson [1] and Myrna J. Simpson [1,*]

[1] Department of Chemistry, University of Toronto, 1265 Military Trail, Toronto, Ontario M1C 1A4, Canada; E-Mails: brian.lankadurai@mail.utoronto.ca (B.P.L); andre.simpson@utoronto.ca (A.J.S.)
[2] Ontario Ministry of the Environment, 125 Resources Road, Toronto, Ontario M9P 3V6, Canada; E-Mails: vasile.furdui@ontario.ca (V.I.F.); eric.reiner@ontario.ca (E.J.R.)

* Author to whom correspondence should be addressed; E-Mail: myrna.simpson@utoronto.ca; Tel.: +1-416-287-7234; Fax: +1-416-287-7279.

Section S1. Earthworm Maintenance Prior to Soil exposure tests.

Eisenia fetida were purchased from The Worm Factory (ON, Canada). They were raised in earthworm bins containing sphagnum peat bedding (Magic Worm bedding; Magic Products; WI, USA) at an approximate temperature of 24 °C. The moisture content of the bedding is 67% water by weight. The earthworms were acclimated for several months to decrease variations in the ^{1}H NMR profile due to differences in diet and other environmental factors [1]. The worms were fed Magic Worm Food (Magic Products; WI, USA).

Section S2. Analysis of soil PFOS concentrations

1. Soil Extraction

PFOS present in soil after two, seven and fourteen days of exposure were extracted in triplicate using the procedure based on Higgins et al. [2]. Homogenized and air-dried OECD soil (1 g) was transferred to a 40-mL polypropylene (PP) vial, to which 10 mL of an acetic acid solution (1%) was added. Each vial was then vortexed, sonicated for 15 min in a preheated bath (60 °C) and centrifuged at 4500 rpm (~1500 g) for 2 min using an International Equipment Company 21000 centrifuge (Fisher Scientific). The acetic acid solution was then decanted into another 40-mL PP vial. A 2.5 mL aliquot of a solvent mixture composed of 90:10 (v/v) methanol and 1% acetic acid in Milli-Q (Millipore Synergy UV, Billerica, MA) water was then added to the original PP vial, which was followed by vortexing, sonication for 15 min at 60 °C and centrifugation at 4500 rpm (~1500 g) for 2 min. The supernatant was again transferred to the second PP vial. The acetic acid wash followed by the methanol/acetic acid extraction was repeated and a final 10 mL wash with acetic acid was performed. All washes and extracts were combined for each sample. The total volume of the extracts and washes was approximately 35 mL.

2. Sample Cleanup

Solid phase extraction (SPE) was performed to concentrate the extracts and to remove the acetic acid, salts, and potential matrix interferences. A 500-mg SUPELCLEAN LC-18 cartridge (SUPELCO, PA, USA) was first conditioned with 10 mL of methanol followed by 10 mL of 1% acetic acid. The extracts were then loaded on to the SPE cartridges that were mounted on a vacuum manifold. The SPE cartridges were then rinsed with 10 mL of Milli-Q water prior to being allowed to dry under vacuum for 2 h prior to elution. PFOS was eluted from the SPE cartridge with 4 mL of methanol and was collected in a 1:1 (v/v) methanol/acetone washed 20 mL glass vials. The eluent was then concentrated to 2 mL under nitrogen and transferred to fresh 20 mL glass vials. The original 20 mL glass vials were then rinsed with 800 µL of methanol. The rinse was combined with the eluent and an additional 1200 µL of 0.01% aqueous ammonium hydroxide solution was added. The extracts were stored at 4 °C until analysis. Prior to analysis the extracts were diluted (10, 000×) into 990 µL of 1:1 methanol/water to which 10 µL of $^{13}C_4$-PFOS aqueous internal standard (750 pg/mL) was added in a 2-mL autosampler glass vial.

3. HPLC-MS/MS Analysis

Analysis of PFOS extracted from the spiked OECD soil was performed using high performance liquid chromatography-tandem mass spectrometry (HPLC-MS/MS). An Agilent 1200 series HPLC was coupled with a 4000 QTRAP triple quadrupole mass spectrometer (Applied Biosystems-MDS Sciex, Concord, ON, Canada). Water and methanol (20 mM Ammonium acetate) were the solvents, which were delivered at a flow rate of 0.5 mL/min. The sample injection volume was 20 µl. The chromatographic separation was obtained using a Kinetex 2.6 µm Phenyl-Hexyl column (4.6 mm i.d. × 100 mm, 2.6 µM; Phenomenex, Torrance, CA). PFOS separation was obtained in 10 mins under gradient conditions, with 65 : 35 methanol:water initial mobile phase, followed by a 0.5 min ramp to 80:20 methanol:water, then a 2 min ramp to 95 : 5 methanol:water, which was held for 4 mins, followed by a 0.5 min ramp back to 65 : 35 methanol:water which was held for 3.50 mins.

The mass spectrometer was operated in negative electrospray ionization multiple reaction monitoring (MRM) mode using previously published methods [3, 4]. MRM transition related parameters were optimized for $PFOS^-$ (m/z = 499) and SO_3F^- (m/z = 99): electrospray voltage = −4500 V, collision energy = −75 V, declustering potential = −103 V, collision exit potential = −10 V, and the dwell time = 200 msec. The $^{13}C_3$-PFOS (Wellington Laboratories, Guelph, ON) internal standard used the same MRM transition related parameters as PFOS for $^{13}C_3$-$PFOS^-$ (m/z=503) and SO_3F^- (m/z = 99). Quantification was performed using the internal standard method with a multi-concentration external calibration curve.

Literature Cited

1. Brown, S. A. E.; Simpson, A. J.; Simpson, M. J. Evaluation of sample preparation methods for nuclear magnetic resonance metabolic profiling studies with *Eisenia fetida*. *Environ. Toxicol. Chem.* **2008,** *27*, 828–836.

2. Higgins, C.P.; Field, J.A.; Criddle, C.S.; Luthy, R.G. Quantitative determination of perfluorochemicals in sediments and domestic sludge. *Environ. Sci. Technol.* **2005,** *39*, 3946–3956.
3. Furdui, V.I.; Helm, P.A.; Crozier, P.W.; Lucaciu, C.; Reiner, E. J.; Marvin, C. H.; Whittle, D. M.; Mabury, S.A.; Tomy, G.T. Temporal trends of perfluoroalkyl compounds with isomer analysis in lake trout from Lake Ontario (1979-2004). *Environ. Sci. Technol.* **2008,** *42*, 4739–4744.
4. Furdui, V.I.; Stock, N.L.; Ellis, D.A.; Butt, C.M.; Whittle, D.M.; Crozier, P.W.; Reiner, E.J.; Muir, D.C.G.; Mabury, S. A. Spatial distribution of perfluoroalkyl contaminants in lake trout from the Great Lakes. *Environ. Sci. Technol.* **2007,** *41*, 1554–1559.

Table S1. PLS-regression model parameters and outputs

Day of Exposure	PLS-components used	R^2X	R^2Y	Q^2Y	*P*-value*
2 day exposure	2	0.59	0.15	-0.10	0.3
7 day exposure	7	0.90	0.74	0.34	7×10^{-4}
14-day exposure	6	0.85	0.74	0.42	2×10^{-5}

* *P*-values were obtained from the normal distribution of the permuted Q^2Y values obtained from a permutation test that involved 400 permutations

Figure S1. PCA loadings plots showing the metabolites that were major contributors to the separation observed in the average PCA scores plot of the *E. fetida* tissue extracts comparing the controls and PFOS exposed earthworms. The abscissa refers to the ^1H NMR chemical shifts (ppm). The loadings plots refer to, two-day exposure (A) PC1 and PC2, (B) PC3 and PC4, seven-day exposure (C) PC1 and PC2, (D) PC3 and PC4, and fourteen-day exposure (E) PC1 and PC2, (F) PC3 and PC4.

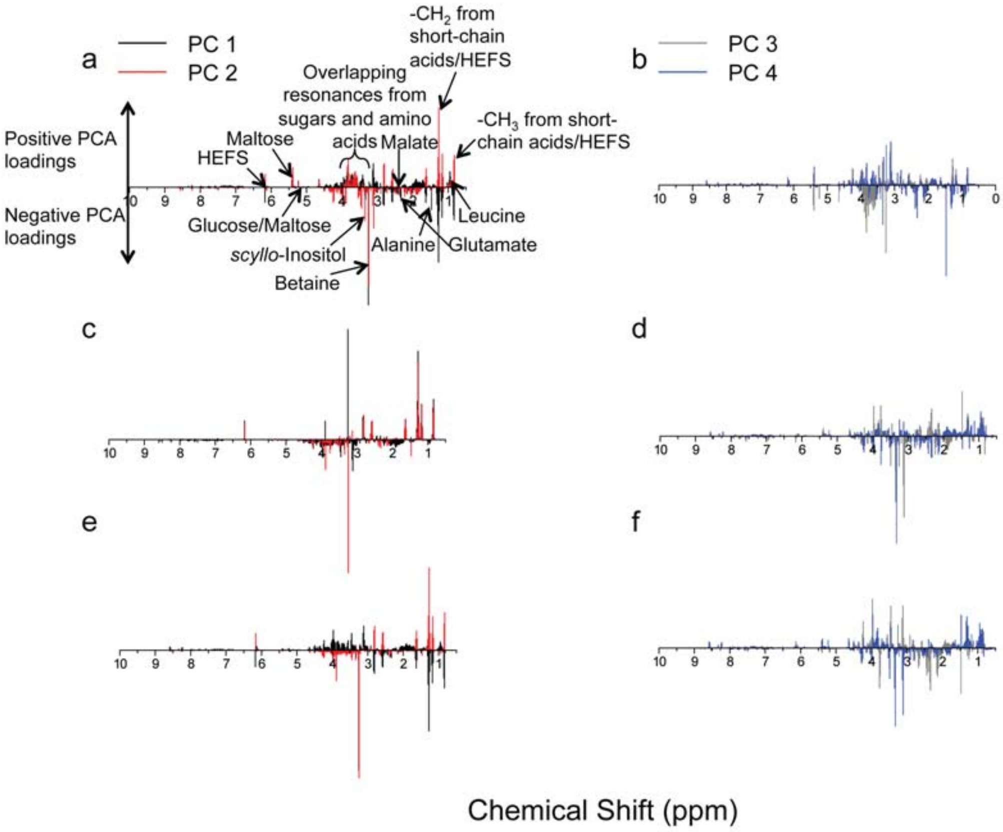

Figure S2 PCA loadings plots showing the metabolites that were major contributors to the separation observed in the average PCA scores plot of the *E. fetida* tissue extracts comparing the controls and earthworms exposed to PFOS for two, seven and fourteen days. The abscissa refers to the ^{1}H NMR chemical shifts (ppm). The loadings plots refer to, (A) PC1 and PC2, and (B) PC3 and PC4

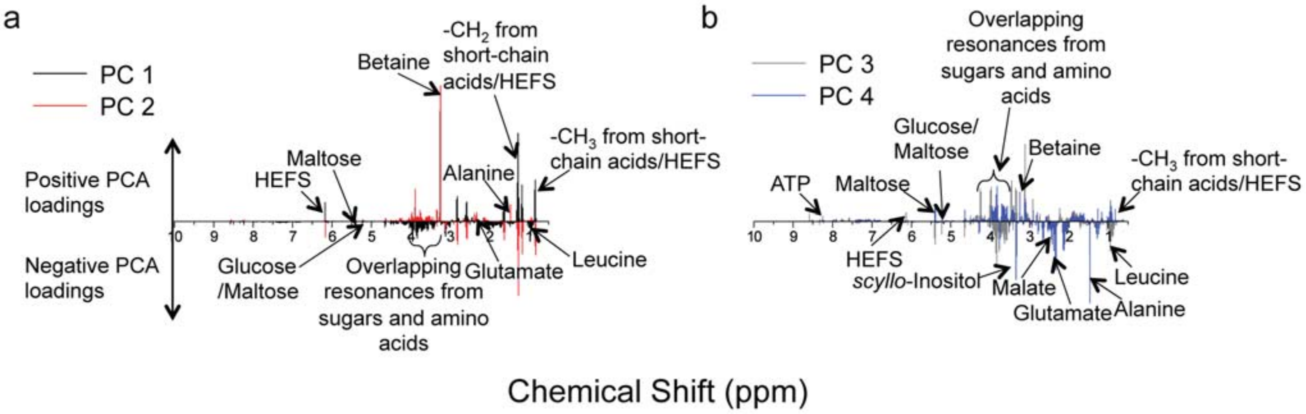

Figure S3. Histograms of Q^2Y values for cross-validated PLS models using the binned ^{1}H NMR spectra as the X-table and random permutations of the PFOS exposure concentrations as the Y variable. Distributions were constructed using 400 permutations of the Y table. The histograms correspond to PLS models constructed for (**A**) two days of exposure, (**B**) seven days of exposure and (**C**) fourteen days of exposure.

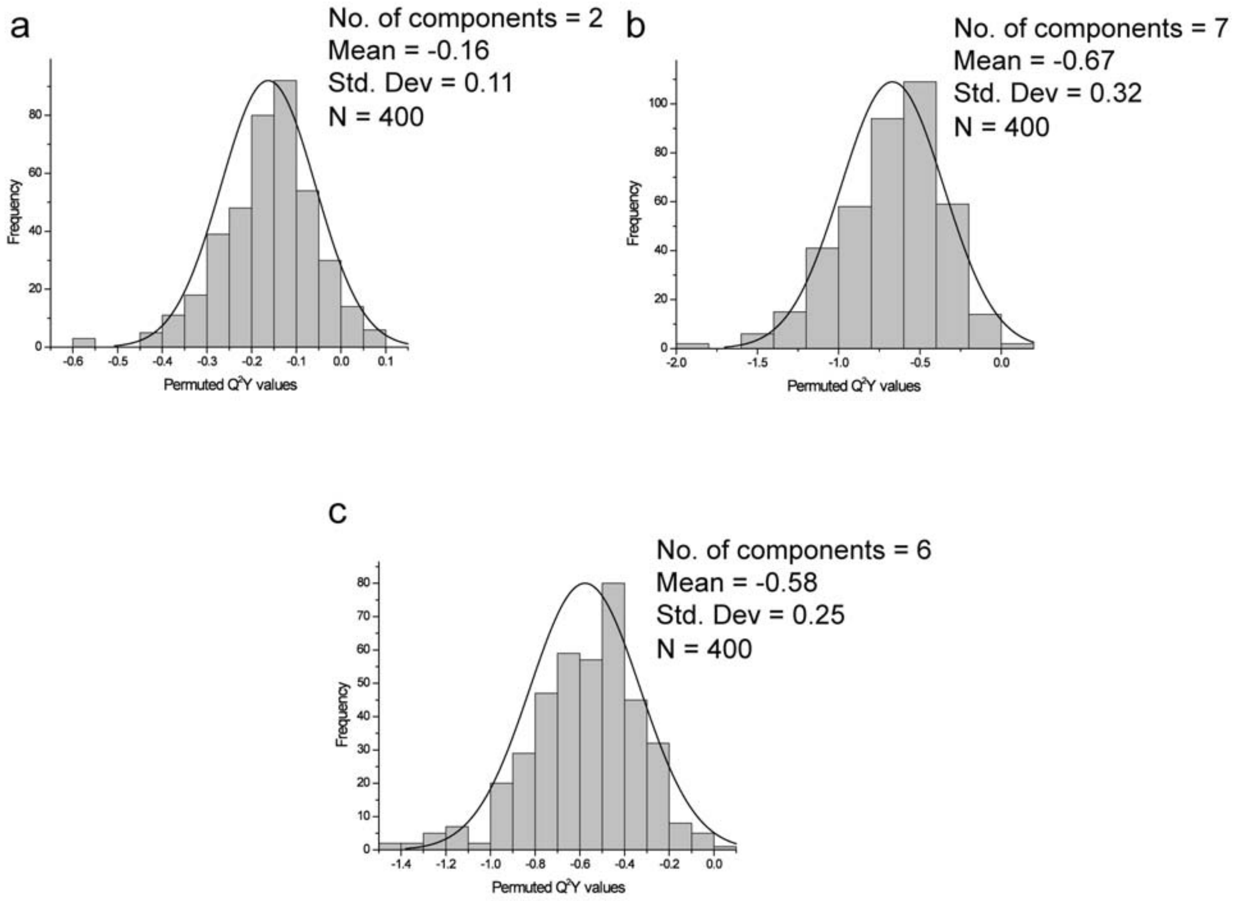

Figure S4. *T*-test filtered ¹H NMR difference spectra of the *E. fetida* tissue extracts obtained by subtracting the mean buckets of the control earthworms from the mean buckets for the PFOS exposed earthworms after two days of exposure and retaining the buckets that were statistically different from the controls at α = 0.05. The *t*-test filtered ¹H NMR difference spectra are shown after PFOS exposure of (**A**) 5 mg/kg, (**B**) 10 mg/kg, (**C**) 25 mg/kg, (D) 50 mg/kg, (E) 100 mg/kg, and (F) 150 mg/kg.

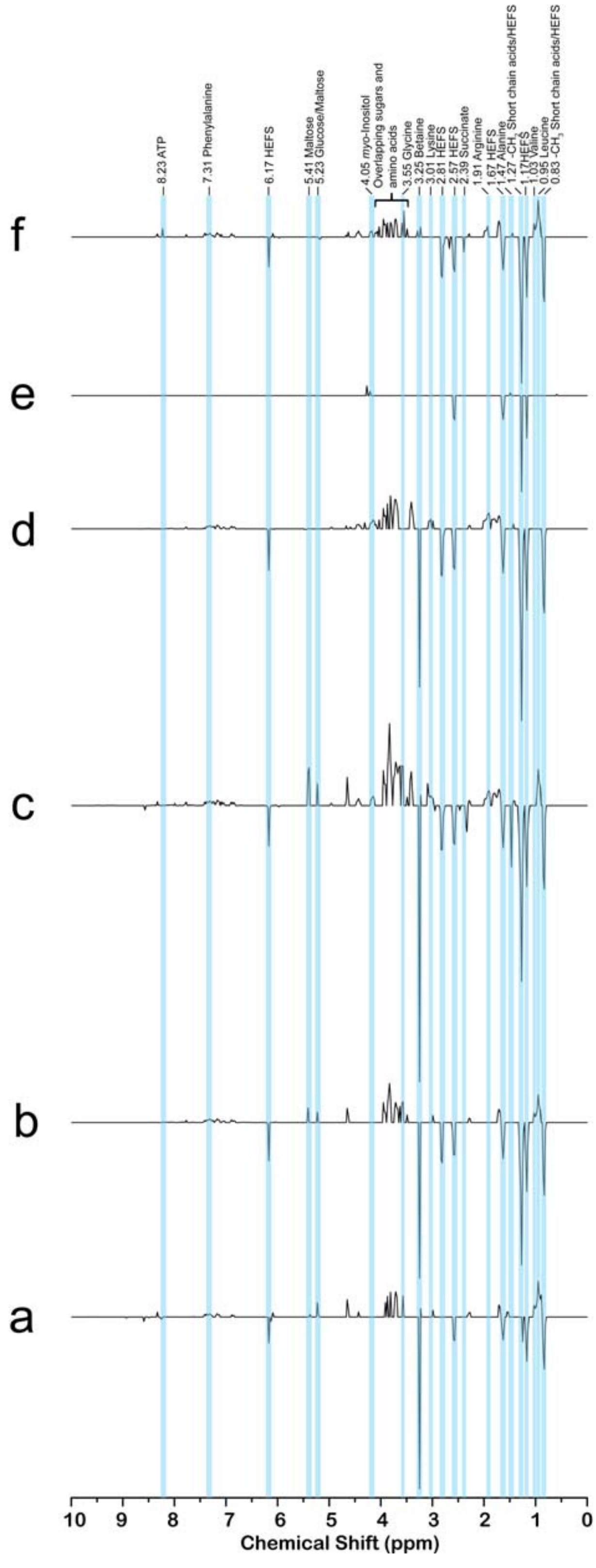

Figure S5. T-test filtered ^{1}H NMR difference spectra of the *E. fetida* tissue extracts obtained by subtracting the mean buckets of the control earthworms from the mean buckets for the PFOS exposed earthworms after seven days of exposure and retaining the buckets that were statistically different from the controls at $\alpha = 0.05$. The t-test filtered ^{1}H NMR difference spectra are shown after PFOS exposure of (**A**) 5 mg/kg, (**B**) 10 mg/kg, (**C**) 25 mg/kg, (**D**) 50 mg/kg, (**E**) 100 mg/kg, and (**F**) 150 mg/kg.

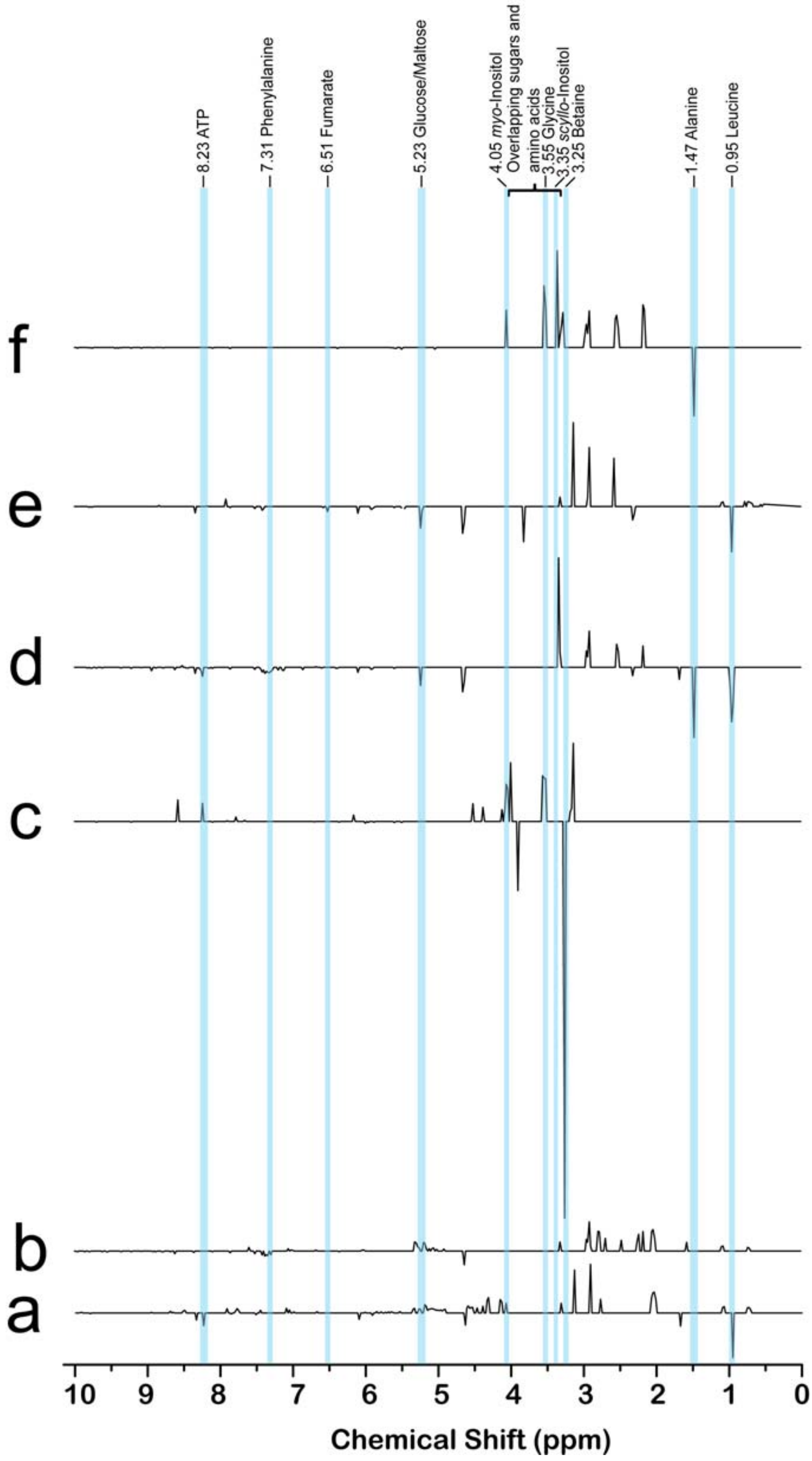

Figure S6. *T*-test filtered ^{1}H NMR difference spectra of the *E. fetida* tissue extracts obtained by subtracting the mean buckets of the control earthworms from the mean buckets for the PFOS exposed earthworms after fourteen days of exposure and retaining the buckets that were statistically different from the controls at $\alpha = 0.05$. The *t*-test filtered ^{1}H NMR difference spectra are shown after PFOS exposure of (**A**) 5 mg/kg, (**B**) 10 mg/kg, (**C**) 25 mg/kg, (**D**) 50 mg/kg, (**E**) 100 mg/kg, and (**F**) 150 mg/kg.

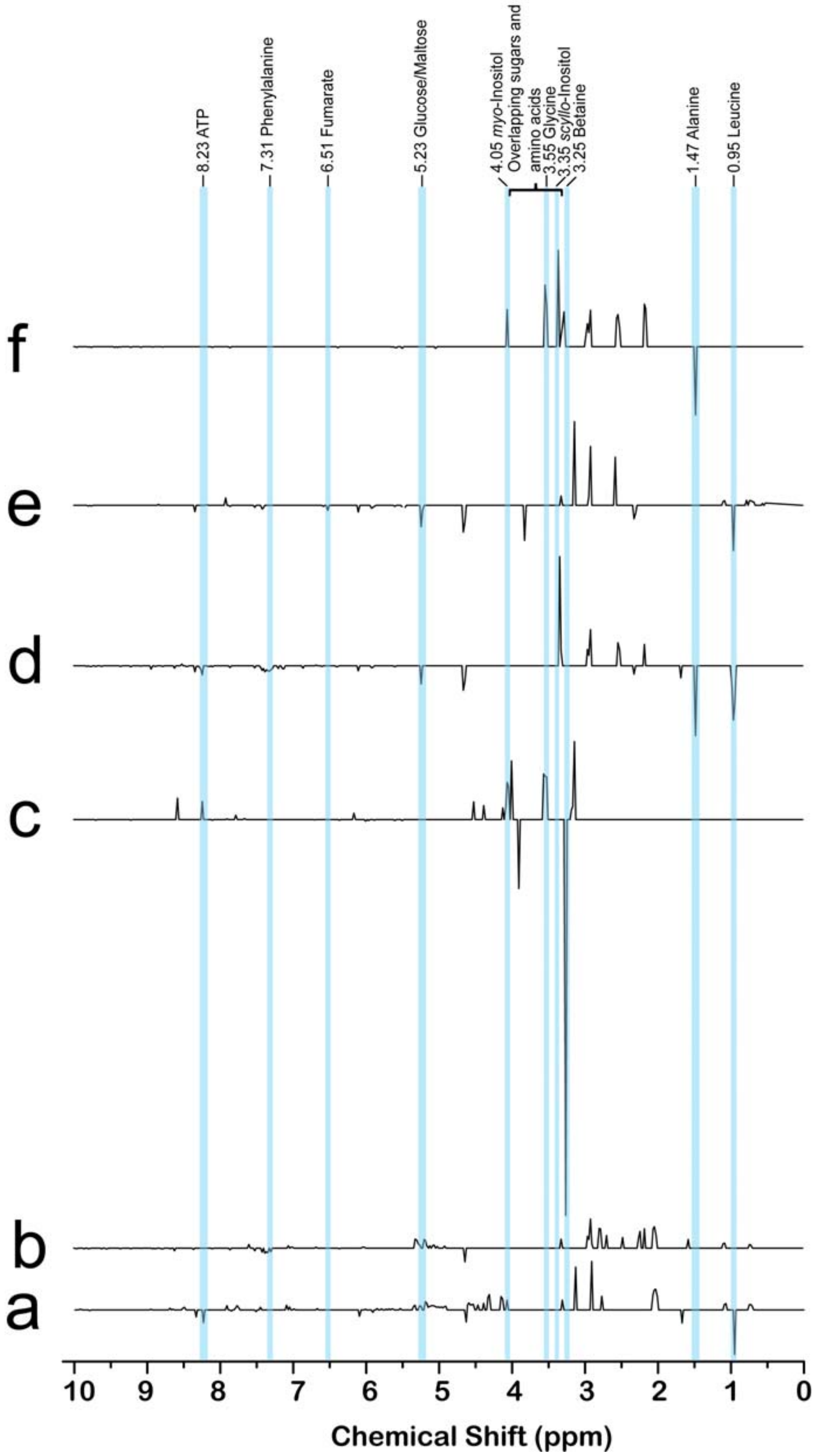

Supplement: Supplementary File 1 — Supplementary (PDF, 478 KB) [file metabolites-03-00718-s001.pdf]
